# Outbreak of acute gastroenteritis in an air force base in Western Greece

**DOI:** 10.1186/1471-2458-6-254

**Published:** 2006-10-17

**Authors:** Eleni Jelastopulu, Danai Venieri, Georgia Komninou, Theodoros Kolokotronis, Theodoros C Constantinidis, Christos Bantias

**Affiliations:** 1 Laboratory of Public Health, School of Medicine, University of Patras, Greece; 2 Laboratory of Hygiene and Environmental Protection, School of Medicine, University of Thrace, Greece; 3 Medical Service, Fighter Wing 117, Hellenic Air Force, Greece

## Abstract

**Background:**

On the 20^th ^September 2005, soldiers and staff at the Air Force base in Western Greece experienced an outbreak of acute gastroenteritis. The purpose of this study was to identify the agent and the source of the outbreak in order to develop control measures and to avoid similar outbreaks in the future.

**Methods:**

A case-control analytical approach was employed with 100 randomly selected cases and 66 controls. Patients completed standardized questionnaires, odds ratios were calculated and statistical significance was determined using χ^2 ^test.

In addition, to identify the source of the infection, we performed bacteriological examination of food samples (included raw beef, cooked minced meat, grated cheese and grated cheese in sealed package) collected from the cuisine of the military unit.

**Results:**

More than 600 out of the 1,050 individuals who ate lunch that day, became ill. The overall attack rate, as the military doctor of the unit estimated it, was at least 60%. The overall odds ratio of gastroenteritis among those who had lunch was 370 (95% CI: 48–7700) as compared to those who didn't eat lunch. Among the symptoms the most prominent were watery diarrhoea (96%) and abdominal pain (73%). The mean incubation period was 9 h and the median duration of the symptoms was 21 h. In the bacteriological examination, *Staphylococcus aureus *was detected in a sample of raw beef (2,000 cfu per g) and in two samples of grated cheese; leftover cheese from lunch (7,800 cfu per g) and an unopened package purchased from the market (3,000 cfu per g).

**Conclusion:**

The findings of this study suggest that the aetiological agent of this outbreak was *S. aureus*. The food vehicle was the grated cheese, which was mixed with the beef and served for lunch in the military unit. This outbreak highlights the capacity of enterotoxin-producing bacteria to cause short term, moderately-severe illness in a young and healthy population. It underscores the need for proper food handling practices and reinforces the public health importance of timely notification of such outbreaks.

## Background

Foodborne diseases are considered a major public health problem because they affect individuals of all ages and socioeconomic status [1]. Despite the use of HACCP programming and hygiene code to ensure the safe preparation of food, many outbreaks of gastroenteritis have been reported, caused by ingestion of pathogens or their toxins in contaminated food ingredients [2-4].

Numerous studies published recently, indicate that most outbreaks of acute gastroenteritis occur in places of mass feeding such as institutions, schools, restaurants and military units [3-8]. The Communicable Disease Surveillance Centre (CDSC) in England and Wales has operated a system of surveillance for general and foodborne outbreaks, according to which the most frequent aetiological agents are viruses (Rotaviruses, Noroviruses), *Salmonella *species, *Clostridium perfringens, Escherichia coli *strains, *Campylobacter jejuni, Listeria, Yersinia *as well as toxins of *Staphylococcus aureus *and *Bacillus cereus *[9-15]. Furthermore, 2004 data obtained from Centers for Disease Control and Prevention (CDC) in USA indicate declines in the incidence of foodborne infections caused by *Campylobacter, Cryptosporidium*, Shiga-toxin producing *E. coli *(STEC) O157, *Listeria, Salmonella *and *Yersinia*. As for the suspected food vehicles, these are usually dairy products, meat-based dishes, sauces and seafood [13-15].

In Greece, recording of outbreaks of foodborne gastroenteritis is limited and there is a considerable lack of recorded epidemiological data. Most cases are not reported, resulting in inadequate monitoring and evaluation of outbreak trends, based on aetiological agents, food vehicles and venues. Thus, prevention and control measures for food safety are difficult to establish.

The present study focused on a reported outbreak of gastroenteritis in an Air Force base in Western Greece, where during an operative training day, soldiers and staff experienced an outbreak of acute gastroenteritis. Following the assessment of descriptive epidemiology, we performed a case-control analytic approach. Using project-designed standardized questionnaires the main objective of the study was to determine the vehicle of the contamination. Furthermore, forced by a public health perspective and taking into account the possible malpractice of employees, we tried to develop control measures in order to prevent future contamination and underline the importance of adherence to high quality hygiene standards for food preparation.

## Methods

### Outbreak investigation

On 20 September 2005, during an operative training day at the Air Force base in Western Greece, soldiers and staff experienced an outbreak of acute gastroenteritis. A case-control analytic approach was utilized, wherein 100 randomly selected subjects out of about 600 taken ill and 66 controls (unaffected soldiers and staff) took part. A questionnaire study was performed 5 days after the outbreak. All subjects completed the same questionnaire during a personal interview by the military doctor. Specifically, there was an effort to interview as many people as possible and to record the same number of cases and controls. Unfortunately, this was not feasible because of the special characteristics of the incident. During that operative training day there were many individuals (soldiers and staff) at the base from other military units as well, who could not be reached afterwards. Thus, it was impossible for the doctor to collect the necessary information from all people who had lunch in the base on 20^th ^September 2005. From personal interviews and the questionnaires collected, those who had not developed any relevant symptom became 'controls', while all others became 'cases'.

Data recorded included setting of the outbreak, dates of onset, frequency and types of symptoms, incubation period, duration of illness and consumption of food items served on the base during the implicated training day.

Odds ratios were calculated for consumption of various food items and statistical significance was determined using the Chi-square (χ^2^) test.

General Headquarters of the Greek Air Force approved the outbreak investigation, which was conducted by the staff of the Laboratory of Public Health of University of Patras (Greece) and the military doctor. Verbal consent was obtained from all study subjects.

### Microbiological analysis

Samples of the food served to the soldiers were taken from the cuisine of the military unit and were transferred to the lab for microbiological analysis. Food samples included a) 1 kg of raw meat (beef), b) 200 g of cooked minced meat (derived from the beef), c) 200 g of grated cheese and d) a sample of the same cheese in its unopened original package. Food items were transported to the laboratory under refrigerated conditions (5 ± 1°C) and analyzed upon arrival. All samples were examined for the detection of *Salmonella *spp.,*S. aureus*, *E. coli *and Heterotrophic Plate Count (HPC) at 22°C and 37°C. Meat samples were further tested for the detection of *Clostridium *spp.

The detection of *Salmonella *spp. was performed according to Standard Methods of the American Public Health Association.

For the other analyses, a homogenate of each sample was prepared by diluting 10 g of sample in 90 ml Peptone Water (Oxoid). For *S. aureus *Baird-Parker's medium (Oxoid) and Manitol salt agar (Oxoid) were used. Surface inoculated plates were incubated at 37°C for 48 h. The typical *S. aureus *colonies were counted and tested for coagulase reaction (Oxoid Staphytect Plus kit). For *E. coli *Tryptone Bile X-Glucuronide medium – T.B.X. (Oxoid) was used and the plates were incubated at 44°C for 24 h. HPC was evaluated using Tryptone Yeast Extract Agar (Oxoid) incubated at 22°C and 37°C for 72 and 48 h, respectively. Finally, TSC agar (Oxoid) was used for the enumeration of *Clostridium *spp. colonies after incubation at 44°C for 24 h under anaerobic conditions.

Water samples from the military unit were also examined applying filtration through nitrocellulose membranes (0.45 μm pore size, 47 mm diameter, Pall-Gelman Laboratory). A 100 ml of each sample was filtered for the determination and enumeration of total coliforms, *E. coli *and *Enterococcus *spp, as imposed by the Greek legislation.

## Results

**Figure 1:**
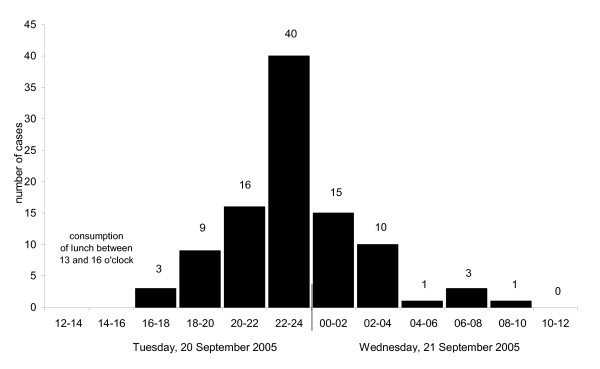
Epidemic curve of cases of acute gastroenteritis in the Air Force Base.

The overall estimated attack rate was at least 60% among the approximately 1050 attendees. The outbreak started abruptly in the late afternoon on 20 September, peaked at midnight and ended about 25 hours later, as demonstrated by the epidemic curve (Figure 1). From the interviews and analysis thereof, it was established that lunch (beef, macaroni, tomato sauce and grated cheese) consumed several hours prior to the onset of the symptoms, was the likely source of the outbreak. The overall odds ratio of gastroenteritis among those who had lunch was 370 (95% CI: 48–7700) as compared to those who didn't eat lunch. Table 1 lists the odds ratio of lunch and dinner consumption.

**Figure 2:**
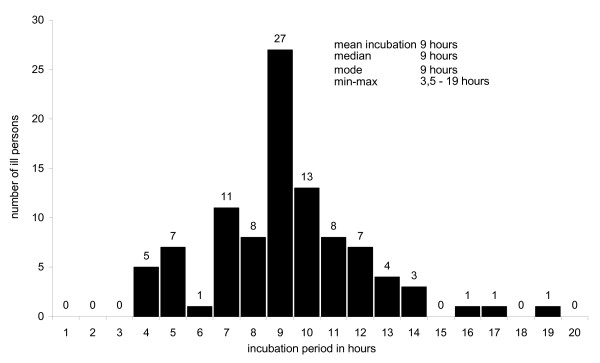
Incubation period of acute gastroenteritis.

**Figure 3:**
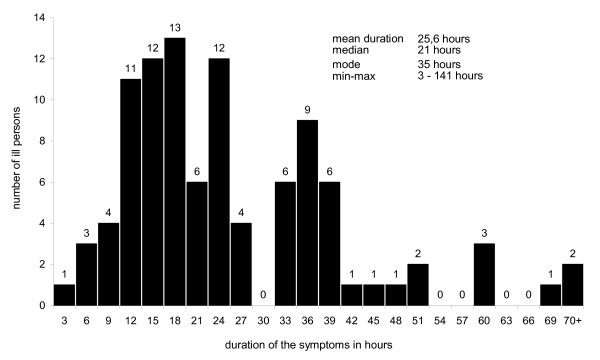
Duration of gastrointestinal symptoms.

The incubation time ranged from 3.5 to 19 h (mean 9 h) (Figure 2). The median duration of the gastrointestinal symptoms was 21 h, ranging from 3 to 141 h (Figure 3).

Among the symptoms reported, the most prominent were watery diarrhoea (96%) and abdominal pain (73%), whereas vomiting and nausea developed in 8% and 7% of the cases, respectively (Table 2). Of the 100 interviewed cases nearly 87% reported their illness as moderate and severe, whereas only 13% reported mild symptomatology.

The results of the microbiological analyses showed that only *S. aureus *was detected in the sample of raw beef (2,000 cfu per g) and in the two samples of grated cheese. In total 7,800 cfu per g were detected in sample "c" (opened bag containing the rest of cheese that had been served to the subjects during lunch) and 3,000 cfu per g in sample "d" (unopened package purchased from the market).

All food samples cultured were negative for the presence of *E. coli*, *Salmonella *spp. and *Colstridium *spp. and HPC ranged in low levels, between 40 cfu and 1500 cfu per g. The analysis of water samples showed negative results for the parameters tested, indicating that the suspected vehicle of the pathogen was among the food items served to the subjects.

## Discussion

The outbreak of acute gastroenteritis, reported on 20 September 2005 on the Air Force base in Western Greece, was originally presumed to be caused by an enterotoxin-producing bacterium. This speculation was based on the short incubation period with abrupt onset, the symptomatology and the short, self-limiting nature of the illness. *S. aureus *was considered to be the most likely cause. Although mortality and longer-term morbidity are uncommon with food poisoning caused by enterotoxin-producing bacteria [18], this outbreak highlights its capacity to cause short term, moderately severe illness in a young and healthy population.

In the present study our main concern was to determine the food vehicle of this reported foddborne gastroenteritis. For this purpose suspected food items were collected and examined. By that procedure we identified the aetiological agent and proceeded with the isolation and withdraw of the contaminated food item. Nevertheless, a limitation of this investigation was the fact that stool samples were not obtained for microbiological diagnosis. The reasons for this omission include the short duration of the symptoms and the overall lack of coordination in the military base, although they are important in suspected foodborne outbreaks, as reported [18]. The data collected from the questionnaires and microbiological analyses lead to the conclusion that *S. aureus*, detected in the cheese was the main aetiological agent of the reported outbreak. As far as the possible cause of the contamination is concerned, the most likely scenario is that the food-handler who packaged grated cheese into the bags in the market was the source, because the pathogen was detected in packaged cheese purchased directly from the market. *S. aureus *colonies were also detected in the beef sample, but that was probably due to cross-contamination from the cheese during lunch preparation.

According to epidemiological studies, a wide range of foods are sources of staphylococci food poisoning, resulting from enterotoxins produced primarily by *S. aureus *[19-21]. Focusing on dairy products, many studies state their association with *S. aureus *which survives well in cheese during production and handling processes [1,16,23,24]. In general, high salt foods like cheese favour the growth of *S. aureus *over other bacteria [18]. The variety of toxins produced by *S. aureus *causes a wide array of disease symptoms, ranking staphylococcal enterotoxins as one of the most common causes of reported food-borne illnesses [16,19,21]. Staphylococcal food poisoning is a persistent cause of gastroenteritis worldwide, especially because Staphylococcal toxin is heat-stable and not destroyed by cooking.

In developed countries *S. aureus *may be more costly to treat than many other food-associated pathogens [21]. The chemical parameters of cheese together with poor hygienic quality favour *S. aureus *proliferation and contamination [23]. These data indicate the necessity of high quality hygienic conditions during manufacturing, storage and marketing. Another critical but often-unrecognised food safety factor is the temperature at which potentially hazardous foods are received [2].

On the other hand, *S. aureus *is an indicator of post process contamination by food handlers, which most likely occurred in our case. Humans are the main reservoir for staphylococci. Hands and the mucous membranes of the nasopharynx are predominant sites of colonization. High rates of carriage and large cells numbers are two major factors that explain the important role of food workers in the epidemiology of staphylococcal food poisoning outbreaks [20]. The majority of the staphylococci inhabiting the nasal fossae of food handlers belong to the species of *S. epidermidis *and *S. aureus *[25].

Reducing the number of foodborne outbreaks will require continued and coordinated efforts by many different agencies, including those of disease surveillance, consumer education and food handling, processing and marketing [26]. To begin with, certain measures should be implemented to correct the faulty food preparation and handling practices. This investigation highlights the importance and severity under which a hazard analysis critical control point (HACCP) system should be applied in all kinds of commercial food services followed by employee adherence. The HACCP system is a systematic approach to the identification, assessment and control of hazards that has been widely accepted as an effective means of ensuring food safety throughout the whole food chain and is compulsory in the European Union [20].

In Greece supervision authorities regarding foodservices are not fully developed and there is a considerable lack of epidemiological evidence, resulting in an unsatisfactory insight in the incident and pathogens of gastroenteritis caused by contaminated food. Thus, limited collected data of outbreaks do not allow the evaluation of outbreak trends and the proper surveillance by the Greek food-hygiene system.

## Conclusion

Information obtained from the questionnaires with the results of microbiological analysis of food items led to the conclusion that the aetiological agent of this outbreak was *S. aureus*. The pathogen was detected and isolated from grated cheese, which served as food vehicle of transmission.

This outbreak highlights the capacity of enterotoxin-producing bacteria to cause short term, moderately-severe illness in a young and healthy population. Foodborne outbreaks like this one recorded in the present investigation should be reported to the associated authorities in order to accomplish appropriate monitoring in time. Thus, food-hygiene services could improve the surveillance of outbreaks, particularly those occurring in institutions or affecting the community at large and protect public health.

## Competing interests

The author(s) declare that they have no competing interests.

## Authors' contributions

EJ had the original idea for the study, developed the questionnaire and organized the case-control study, carried out the statistical analysis, formed the layout of the manuscript and wrote the first draft of the manuscript.

DV carried out the microbiological analysis, contributed on interpretation of the results and co- wrote the final manuscript.

GK carried out the microbiological analysis and contributed to the preparation of the manuscript.

TK participated in the data collection and interpretation.

TCC contributed in the data interpretation and the final manuscript.

CB carried out and supervised the data collection at the air force base

All authors read and approved the final manuscript.

## Pre-publication history

The pre-publication history for this paper can be accessed here:



## Figures and Tables

**Table 1:** Case-control study and odds ratios

**Meals**	**Cases (n = 100)**		**Controls (n = 66)**		**Odds Ratio**	**95% CI**
			
	Yes	No	Yes	No		
Lunch on 20.9.2005 (complete or partly)	99	1	14	52	370	48 – 7700
Lunch complete	79	21	7	59	32	12 – 90
Dinner on 20.9.2005	27	72	44	22	0.8	0.4 – 1.5

**Table 2:** Prevalence of symptoms among the 100 soldiers with gastroenteritis

**Symptom**	**Patients (n)**
Diarrhoea (watery)	96
Abdominal pain	73
Vomiting	8
Nausea	7
Headache	6
Fatigue	4
Fever	3
